# Barriers and facilitators to recruiting older adult care home residents into clinical trials of medicines and vaccines: a scoping review

**DOI:** 10.1093/ageing/afag077

**Published:** 2026-04-09

**Authors:** Selvarani Subbarayan, Imogen Smith-Dodd, Gabriel Nicolson, Jennifer K Burton, Janet T Scott, Seshadri S Vasan, Susan D Shenkin, Roy L Soiza, The WATCH Consortium, Adam Lee Gordon, Charis A Marwick, Shaun Treweek, Carrie Stewart, Maria Drummond, Rosalie Ashworth, Harish Nair, Laura Shallcross, Mary Joan MacLeod, Terry J Quinn, Robert A Lockhart, Samantha Carmichael, Sandra Prew, Bernie McInally, Kirsty Hynes, Lynne Anderson, Elaine Hodge

**Affiliations:** Ageing Clinical and Experimental Research Group, The Institute of Applied Health Sciences, School of Medicine, Medical Sciences and Nutrition, University of Aberdeen, Aberdeen, UK; Aberdeen Royal Infirmary, NHS Grampian, Aberdeen, UK; Ageing Clinical and Experimental Research Group, The Institute of Applied Health Sciences, School of Medicine, Medical Sciences and Nutrition, University of Aberdeen, Aberdeen, UK; Ageing Clinical and Experimental Research Group, The Institute of Applied Health Sciences, School of Medicine, Medical Sciences and Nutrition, University of Aberdeen, Aberdeen, UK; Academic Geriatric Medicine, School of Cardiovascular and Metabolic Health, University of Glasgow, Glasgow, UK; Research, Development and Innovation, NHS Highland, Inverness, UK; School of Medicine, Medical Sciences and Nutrition, University of Aberdeen, Aberdeen, UK; Centre for Virus Research, University of Glasgow, Glasgow, UK; Aberdeen Royal Infirmary, NHS Grampian, Aberdeen, UK; School of Medical and Health Sciences, Edith Cowan University, Joondalup, Australia; Ageing and Health, and Advanced Care Research Centre, Usher Institute, College of Medicine and Veterinary Medicine, The University of Edinburgh, Edinburgh, UK; Ageing Clinical and Experimental Research Group, The Institute of Applied Health Sciences, School of Medicine, Medical Sciences and Nutrition, University of Aberdeen, Aberdeen, UK; Aberdeen Royal Infirmary, NHS Grampian, Aberdeen, UK

**Keywords:** care homes, nursing homes, randomised controlled trials, vaccine trials, barriers and facilitators, care home residents, older people

## Abstract

**Introduction:**

Excluding Care Home (CH) residents from clinical trials of medicines and vaccines has led to an evidence gap. Findings from younger adults may not be generalisable to CH residents with frailty and dementia. Therefore, it is essential to evaluate pharmacological treatments directly in CH residents to assess their safety and efficacy.

**Methods:**

As part of the Widening Access to Trials in Care Homes project, we conducted a scoping review using Joanna Briggs Institute methodology to identify barriers and facilitators to recruiting CH residents into clinical trials of medicinal products. We searched EMBASE, MEDLINE, PsycINFO, CINAHL, and the Cochrane Library (1990 to January 2025). Quantitative data on screen failures and dropouts, and qualitative data on trial teams’ real-world experiences and stakeholder perspectives were extracted, synthesised into themes and presented as descriptive summaries.

**Results:**

From 14,301 records, eight articles were included (2002 to 2021) from the USA (N = 4), UK (N = 3) and France (N = 1). Six were RCTs, and two were a qualitative sub-study and a questionnaire survey. Screen failure rates ranged from 18% to 96%. Identified themes of barriers and facilitators were study design, selection criteria, recruitment methods, data collection methods, participant retention, CH resident and family-related factors, CH facility and staff-related factors, healthcare professional involvement, logistical and operational factors, consent-related factors, ethical and regulatory considerations, insurance and trial costs, and generalisability of trial results.

**Conclusions:**

Our review specifically identified key barriers and facilitators to recruiting CH residents into pharmacological trials. These findings can guide trial planning and support evidence-based care for this vulnerable population.

## Key points

- Exclusion of care home residents in trials creates an evidence gap in the safety and efficacy of pharmacological interventions.
- Eight studies reported trial teams’ real-world experience of conducting randomised controlled trials in care homes.
- We identified 13 themes of barriers and facilitators to recruiting care home residents into trials of medicines and vaccines.

## Introduction

The COVID-19 pandemic highlighted the substantial underrepresentation of Care Home (CH) residents in randomised controlled trials (RCTs) of pharmacological interventions, including medicines and vaccines. Despite this, CH residents continue to be largely excluded in clinical trials [1–4]. This exclusion has led to a significant knowledge gap regarding the safety and efficacy of new pharmacological interventions for this group [1]. Although some progress has been made in including older adults in pharmaceutical trials, the results from healthier, younger older adults may not be generalisable to CH residents with frailty, dependency, cognitive impairment and polypharmacy due to differences in pharmacokinetics and pharmacodynamics [1, 2, 5, 6].

The evidence base for many pharmacological interventions in CH residents with complex health and social care needs is limited [3]. Therefore, it is crucial to test new pharmacological interventions specifically in the CH population to assess the benefit–risk ratio for this vulnerable group [1, 2]. However, recruiting CH residents to clinical trials presents significant challenges as they may be living with cognitive impairment and reduced mobility [7].

Previous reviews have explored the barriers and facilitators to conducting clinical research in CH settings. Nonetheless, they have largely focused on non-pharmacological or varied intervention types, trial methodologies, research culture, and diverse study designs, providing limited evidence on recruitment challenges specific to RCTs of pharmacological interventions [8–13]. Clinical trials involving pharmacological interventions are often more challenging than other types of clinical trials [14]. A cross-sectional survey study reported that the main barriers for older adults to participate in clinical trials were fear of side effects, polypharmacy, mobility limitations, and sensory impairments [15]. Therefore, it is important to understand the key barriers and identify effective facilitators to address them. The Widening Access to Trials in Care Homes (WATCH) project [16] was established in the UK to develop best practice guidance for improving the inclusion of CH residents in vaccine trials. The first WATCH review identified all global vaccine trials that had recruited CH residents [17]. The aim of this scoping review was to identify papers that reported barriers and/or facilitators to recruiting older CH residents into clinical trials of medicines and vaccines.

## Methods

The scoping review was undertaken following the Joanna Briggs Institute methodology for conducting scoping reviews [18, 19]. The protocol was registered on the Open Science Framework (https://osf.io/y64kq) and reported in accordance with the Preferred Reporting Items for Systematic reviews and Meta-Analyses extension for Scoping Reviews (PRISMA-ScR) checklist [20]. The completed checklist is provided in Appendix 1.

### Search strategy

A comprehensive search strategy, guided by the Population, Concept, Context framework was developed using the expertise of the review team, CH setting-specific terms from previous studies [8, 21, 22], and search filters recommended by the InterTASC Information Specialists’ Sub-Group Search Filter Resource [23] and Cochrane Handbook [24]. The search strategy was further refined in consultation with an experienced librarian. A variety of terminologies are used in the literature to describe CH settings and usage varies significantly across countries. For consistency, we used the term ‘care home’ throughout this review except where alternative terms were used by study authors.

The search was conducted on 19 January 2025 by one author (SS) across five databases: EMBASE, MEDLINE, PsycINFO, CINAHL, and the Cochrane Library from 1990 to January 2025. Detailed database search results are provided in Appendix 2 (Supplementary Tables S1–S5). No language restrictions were applied. Grey literature searching was not carried out, however reference checking and forward citation searching of the included articles were performed by two authors (ISD and GN). All results from the databases were exported to Rayyan-Intelligent Systematic Review web-tool (https://www.rayyan.ai/) where duplicates were removed before screening articles.

### Inclusion and exclusion criteria

We included quantitative and qualitative studies, as well as other relevant sources that reported on barriers and facilitators for recruiting CH residents to clinical trials of pharmacological interventions (medicines and vaccines). Review articles were included only for reference checking to find any additional primary sources. Exclusion criteria were mean age of residents <65 years, articles reporting non-pharmacological therapies and generic challenges in CH research without specifying RCTs, non-randomised trials, and trials in non-CH settings (e.g. sheltered housing).

### Study selection

Two authors (ISD and GN) independently screened titles and abstracts and shortlisted the articles for full text review. Three authors (ISD, GN and SS) independently assessed the full text for eligibility. Disagreements during the study selection process were resolved by consensus, in consultation with a senior team member (RLS).

### Data extraction and analysis

Two reviewers (ISD and GN) independently extracted data using a Microsoft Excel sheet, and a third reviewer (SS) verified and reconciled the data. Data were extracted on study and participant characteristics, CH details, ethical aspects, and funding. Quantitative data on screen failures and dropouts with reasons were extracted where reported. Qualitative data on challenges experienced by the research team and the strategies implemented during the trial conduct were extracted. Additionally, barriers and facilitators reported in other sources of evidence (e.g. qualitative study) related to clinical trials of pharmacological interventions in the CH population were also collected. If a study recruited from multiple settings (e.g. hospital and CHs), only data relevant for CH settings were extracted. The data extraction form is provided in Appendix 3.

Quantitative data are presented as descriptive summaries and in tables. Qualitative data were categorised into two broad descriptive themes: barriers and facilitators based on trial teams’ real-world experience of conducting RCTs, and the views of healthcare professionals, trial teams, CH staff, residents, and relatives regarding clinical trials of pharmacological interventions. These identified barriers and facilitators were then synthesised into major themes, with relevant factors compiled under each theme. Qualitative findings are reported as descriptive summaries and are also presented in tables and figures.

## Results

**Figure 1:**
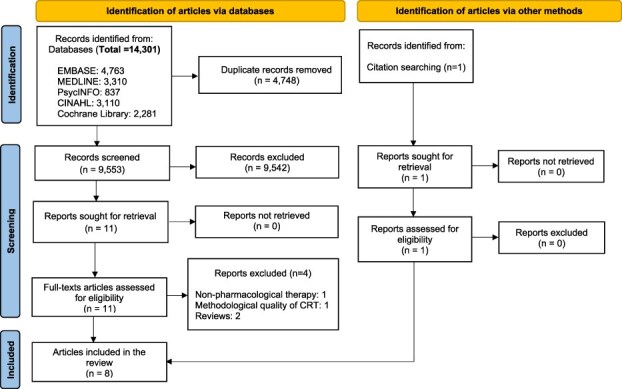
PRISMA flow diagram of the study selection process [51]. CRT: Cluster Randomised Trial.

The database search retrieved 14,301 records. After removing duplicates, 9,553 titles and abstracts were screened. Eleven full-text articles were identified through database searches and one via citation searching, of which eight were included in the review. The PRISMA flow diagram is provided in Figure 1.

### Study characteristics

All included studies were published between 2012 and 2021, except one from 2002. All were conducted in high-income countries: the USA (N = 4), UK (N = 3) and France (N = 1). Of the eight articles, six [25–30] were RCTs of pharmacological interventions, one [31] was a qualitative sub-study and one used a questionnaire survey [32]. The RCTs evaluated medications for osteoporosis (N = 2) [25, 28], antibiotic-associated diarrhoea [27], COVID-19 post-exposure prophylaxis [29], and dementia [30], with one assessing the influenza vaccine [26]. Four trials investigated licensed medicines or vaccines. The COVID-19 trial [29] evaluated an unlicensed Investigational Medicinal Product (IMP), and a probiotic trial intending to test a commercially available food supplement was not implemented [27].

A total of 1,085 CH residents were recruited across five RCTs [25, 26, 28–30], with 14 in the qualitative sub-study [31] and 84 in the questionnaire survey [32]. One RCT did not recruit any participants [27]. Sample sizes in five trials ranged from 17 to 310 and participating CHs from one to 74. Four studies reported a mean age of >80 years and > 71 years in one. Three studies included ≥60% female participants and one osteoporosis trial enrolled only women. Four studies reported that 88% to 97% of the participants were White. CH size was reported in two studies: one trial [25] recruited from nine CHs with 101 to 391 residents, and another [30] from a single large nursing home. Two studies [25, 26] indicated that CHs provided both residential and nursing care. None reported CH ownership. Four trials were publicly funded, and one [29] was industry sponsored. Ethics committee approval was reported in four RCTs [25–27, 29] and the qualitative sub-study [31]. The study characteristics are summarised in Table 1.

**Table 1:** Characteristics of included studies

Author	Title	Country	Study design/ Type of evidence	Disease condition	Study population/ Setting	CHs (N)	Sample size	Mean age (years)	Female (%)	Intervention versus Comparator
**Randomised Controlled trials**										
Greenspan et al. 2012 [25]	Lessons Learned from an Osteoporosis Clinical Trial in Frail Long Term Care Residents	USA	Double-blind, placebo-controlled, RCT: Lessons learned (ZEST trial)	Osteoporosis	Nursing homes and assisted living facilities	9	181	85.3 ± 5.1	NR	Zoledronic acid (5 mg; single dose; 45 min IV infusion) (licensed) vs Placebo (normal saline)
Whelan et al. 2013 [26]	Impact of the demand for ‘proxy assent’ on recruitment to a randomised controlled trial of vaccination testing in care homes	UK	RCT: Analyses of participants included and excluded in the FEVER trial	Influenza	Care homes	26^a^	277	82.0 ± 8.5	67%	Trivalent influenza vaccine and a booster dose vs trivalent influenza vaccine^a^ (licensed)
Shepherd et al. 2015 [27]	Setting up a clinical trial in care homes: challenges encountered and recommendations for future research practice	UK	RCT: Authors experiences of setting up a CTIMP (PAAD study)	Antibiotic-associated diarrhoea	Planned in Care homes but the trial was not implemented.	24^b^	Planned sample size 400: did not recruit.	NA	NA	A probiotic preparation (VSL#3), a commercially available food supplement vs Placebo
Kotlarcyzk et al. 2020 [28]	Characteristics of older adults who do or do not proceed to randomization in a clinical trial	USA	RCT: Reasons for study withdrawal	Osteoporosis	Long-term care setting	NR	310	80.6 ± 7.8	100%	Zoledronic acid (licensed); Comparator: NR
Knorr et al. 2021 [29]	Innovative clinical trial design and delivery: a phase 3 COVID-19 post-exposure prophylaxis study in skilled nursing and assisted living facilities (BLAZE-2)	USA	Double-blind, placebo-controlled RCT: Innovative trial design and operational model	SARS-CoV-2 infection and COVID-19	Skilled nursing and assisted living facilities, including long-term care and nursing home facilities	74^a^	300 residents and 666 CH staff	Mean age of residents: 71.8 ± 12.7	59.7%^a^	Bamlanivimab (4200 mg, single IV infusion at baseline) (unlicensed) vs Placebo (0.9% sodium chloride solution)
Cohen-Mansfield 2002 [30]	Recruitment Rates in Gerontological Research: The Situation for Drug Trials in Dementia May Be Worse Than Previously Reported	USA	Double-blind study in dementia: Recruitment issues experienced by the research team	Dementia	Nursing home residents	1	17	NR	NR	An approved drug, details of intervention and compactor were not reported
**Other types of evidence**										
Wood et al. 2013 [31]	Consent, including advanced consent, of older adults to research in care homes: a qualitative study of stakeholders’ views in South Wales	UK	Embedded qualitative sub-study prior to the RCT: Views and experiences of CH residents, relatives, CH staff & GPs	Antibiotic-associated diarrhoea	Care homes participated in the RCT (PAAD study)	11	Individual interviews: 38 (14 residents, 14 relatives, & 10 GPs) Focus groups: 19 CH staff	NR	NR	NA (face-to-face interviews)
Bloch and Charasz 2014 [32]	Attitudes of Older Adults to Their Participation in Clinical Trials: A Pilot Study	France	A Pilot study: Questionnaire survey, administered in-person	NA	Living at home, long-term care units and hospitalised for acute illness	NR	Total: 150 (n = 84, 56% from long-term units)	84 (aged between 70 and 101)	69%	NA (questionnaire survey)

^a^Data obtained from the original trial publication;

^b^Planned to recruit from 24 nursing homes but the trial was not implemented; RCT: Randomised Controlled Trial; CTIMP: Clinical Trial of an Investigational Medicinal Product; ZEST: Zoledronic acid in frail Elders to STrengthen bone study; FEVER: Flu-Effect of Vaccine in Elderly Residents; PAAD: Probiotics for Antibiotic Associated Diarrhoea in Care Homes; CHs: Care Homes; GPs: General Practitioners; IV: Intravenous; NR: Not Reported; NA: Not Applicable.

### Screen failure and dropouts

Screen failure rates, reported in four studies, ranged from only 18% to 96%. Two studies [25, 26] reported approximately 70%, and the highest rate 96% was seen in a dementia trial [30]. Major reasons for screen failure were residents declining study participation (n = 254, 47%) [25], relatives’ unavailability or refusal (n = 310, 45%) [26] and inability to complete MMSE (n = 127, 34%) [30]. Dropout rates were only reported in two studies at 8% [25] and 71% [30]. Detailed information on screen failures is presented in Table 2.

**Table 2:** Screen failure and reasons

Author	Total screened (*n*)	Total recruited (*n*)	Screen failure (*n* (%))	Reasons for screen failure (*n*, %)
Greenspan et al. 2012 [25]	733	181	536 (73.1%)	Declined (254, 47.4%) Did not meet study criteria (161, 30%) Relatives refused or relatives couldn’t be contacted (112, 20.9%) Died during screening (9, 1.7%)
Whelan et al. 2013 [26]	968	277	691 (71.4%)	Relatives refused or couldn’t be contacted (310, 44.9%) Team considered residents without capacity likely to physically resist study procedures (146, 21.1%) Did not meet study criteria (97, 14.0%) Declined (79, 11.4%) Residents not available (18, 2.6%)
Kotlarcyzk et al. 2020 [28]	376	310	66 (18.0%)	Counselling from a family member or physician (n = 20, 30%) Health concerns (n = 12, 18%) Lack of time (n = 11, 17%) Age (n = 7, 11%)
Cohen-Mansfield 2002 [30]	386	17	369 (95.6%)	MMSE score ≤ 5 (147, 39.8%) MMSE not available, refused or unable to complete MMSE (127, 34.4%) Medical reasons (69, 18.7%) Refused consent (26, 7.0%)

Abbreviation: MMSE: Mini Mental State Examination.

### Barriers and facilitators

**Figure 2:**
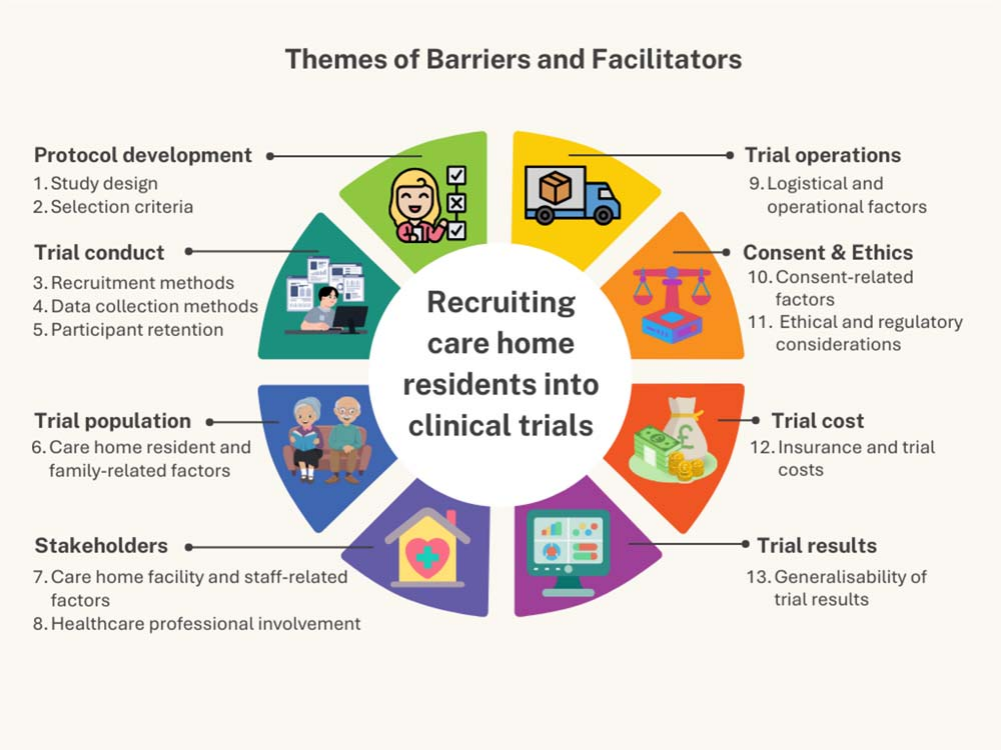
Themes of barriers and facilitators to recruiting care home residents into clinical trials of medicines and vaccines.

Barriers and facilitators to recruiting CH residents into clinical trials were categorised into 13 major themes: study design, selection criteria, recruitment methods, data collection methods, participant retention, CH resident and family-related factors, CH facility and staff-related factors, healthcare professional involvement, logistical and operational factors, consent-related factors, ethical and regulatory considerations, insurance and trial costs, and generalisability of trial results. Barriers and facilitators are presented together, as the same 13 themes apply to both and are interpreted as barriers or facilitators depending on context. The major themes and their associated factors are summarised in Table 3**,** and an infographic aligning these themes with different stages of trial conduct is presented in Figure 2.

**Table 3:** Barriers and facilitators to recruiting care home residents into clinical trials of medicines and vaccines

	Barriers	Facilitators
Protocol development	**Study design**	
	• Pharmacological intervention trials are often perceived as high-risk, unsafe and offering no direct benefit to participants • Difficulty contacting relatives and delays in enrolling CHs within short timeframe	• Design a flexible, low-risk study protocol. • Provide expert recommendations of risks and benefits in plain English • Allow sufficient time in study plans to accommodate CH residents needs
	**Selection criteria**	
	• Strict eligibility criteria • Exclusions based on medical reasons	• Simplify the eligibility criteria • Limit exclusions to essential safety concerns or contraindications
Trial conduct	**Recruitment methods**	
	• Traditional recruitment methods are not suitable for CH settings • Medically qualified professional required at each CH to confirm eligibility and sign consent	• Use CH social events, flyers, newsletters and appoint a study champion at CH • Collaboration with CHs and involve GPs as research partners for recruitment and prescribing
	**Data collection methods**	
	• Challenges for participants to travel to hospital or clinic sites • Safety data may be unreliable due to CH residents’ cognitive impairment	• Use equipped mobile research units to collect data onsite in CHs • Flexible or remote data collection and reduce blood sampling • Use electronic surveillance and multiple data sources to gather safety data
	**Participant retention**	
	• Living with frailty and unstable chronic conditions • High withdrawal rates due to memory impairment	• Provide gift or appreciation cards, appoint resident champions and involve relatives and physicians during recruitment • Calculate sample size to account for high attrition rates
Trial population	**CH resident and family-related factors**	
	• High refusal due to concerns regarding CH residents’ health, frailty or potential harm from trial participation • Difficulty understanding and retaining study information. • Distrust and feeling of ‘being a guinea pig’ • Relatives making decisions based on their own views rather than the resident’s wishes. • Capacity assessments perceived as stressful	• Participation driven by altruism or a desire to support research and the CH community • Explain risks and benefits in simple, non-technical language • Conduct informal capacity assessments or have assessments performed by senior CH staff
Stakeholders	**CH facility and staff-related factors**	
	• Diverse backgrounds and training of CH staff hinder research engagement • Delays in recruiting CH facilities • Study tasks increase workload to CH staff with already high demands • High staff turnover • Cultural factors and language barriers among staff	• Initial meetings with CH professionals, followed by physicians to explain the study and obtain permission • Place research team at CHs to conduct all study procedures and reduce staff burden • Provide training and professional development opportunities to CH staff.
	**Healthcare professional involvement**	
	• Non availability of physicians at CHs to make medical decisions • Hesitancy of healthcare professionals to include adults lacking capacity in trials • Involving GPs as trial sites alongside CHs is complex due to contracts and approvals, especially in the UK	• Recruit GPs formally as research partners in addition to CHs as trial sites to help medical decisions and overall trial conduct • Implement strategies to reduce GPs burden, and simplify contract processes • Train trial teams on CH trials and on effective communication with older adults • Involve a multidisciplinary trial team

*(Continued)*

#### Study design

Pharmacological intervention trials are often perceived by residents and relatives as high-risk, harmful to health, and offer limited direct benefit which may contribute to high refusal rates [30–32]. Additionally, difficulties in contacting relatives within the recruitment timeframe, and substantial delays in enrolling CHs hindered participant recruitment, sometimes resulting in early trial termination [25–27].

Strategies to improve trial conduct included designing flexible and low-risk protocols [25, 29]. One study suggested involving an independent expert panel to assess risks and benefits and presenting their recommendation in plain English to support informed decision-making by CH residents, relatives, and staff [26]. Allocating sufficient time in study plans to accommodate CH-specific needs, residents’ health status, and operational constraints is essential for enhancing recruitment [25, 29, 30].

#### Selection criteria

Strict exclusion criteria, particularly those based on medical reasons have led to high exclusion rates of potential residents [25, 30]. Two studies suggested relaxing eligibility criteria, limiting exclusions only to essential safety concerns or contraindications to improve recruitment [25, 30]. Two trials simplified the eligibility criteria by removing upper age limits, and including residents with cognitive impairment, immobility, multiple chronic conditions and polypharmacy to achieve more efficient recruitment [25, 29].

#### Recruitment methods

Traditional recruitment methods are often impractical in CH settings [25] and regulatory requirements necessitating a medically qualified person at each CH to confirm eligibility and prescribe investigational products further hinder recruitment [27]. To address these challenges, one trial implemented a range of CH-specific strategies including social events (e.g. bingo games, meet and greets, information gatherings), flyers, word-of-mouth, newsletters to relatives, and a peer advocate [25]. Collaboration with CH professionals is essential for identifying eligible participants, and supporting informed consent [25, 31]. Furthermore, involving General Practitioners (GPs) as research partners can assist with eligibility assessment, prescribing, and medical decision-making [27, 32].

#### Data collection methods

One trial reported ‘patient reluctance and transportation challenges to travel to a hospital or clinic’ and unreliability of self-reported safety outcomes due to cognitive impairment as barriers [25]. To address this, investigators used a Mobile a Research Unit (MRU) equipped to conduct study procedures and collect data directly at CH sites. However, this limited outcomes to those feasible to measure within the CH setting [25]. Additional strategies implemented by trials include flexible data collection locations (CH or hospital), adaptable schedules for residents’ changing status, remote data collection, and less frequent blood sampling [25, 29]. To improve safety data capture, one trial implemented several methods: an electronic surveillance system, place study stickers in CH records and ask CH staff to notify study team, daily nurse visits post-IMP administration, regular CH record reviews and reports from relatives [25].

#### Participant retention

Factors unique to CH residents including frailty, and unstable chronic conditions can significantly affect study completion [30]. Mild cognitive impairment at enrolment may cause misunderstandings about participation, increasing withdrawal later. Temporary loss of capacity is also common, often due to infection-related delirium [31]. To improve retention, one trial provided small gifts and appreciation cards, and appointed ‘resident champions’ to encourage continued participation. This trial also accounted for a 30% 1-year attrition rate in sample size calculation [25]. Another trial recommended involving relatives and physicians during recruitment to improve retention [28].

#### CH resident and family-related factors

CH residents were concerned that they were too old or frail to participate in research and worried about potential harm. These concerns, frequently shared by relatives, healthcare professionals and CH staff discouraged participation even when residents had capacity and were willing [30–32]. Residents were also less willing to participate in trials offering no personal benefit, sometimes expressing distrust and likening participation to ‘being a guinea pig’ [25, 32]. Additionally, the capacity assessment process distressed some residents, leading some to decline participation [26].

**Table 3:** Continued

	Barriers	Facilitators
Trial Operations	**Logistical and operational factors**	
	• Transportation challenges for participants • Lack of essential research infrastructure in CHs	• Research staff to conduct visits at CHs • Use equipped mobile research units as extensions of investigator sites
Consent and Ethics	**Consent-related factors**	
	• Complex terminology in information sheets discourages participation • Cognitive impairment makes understanding study information difficult. • Lengthy consent process is overwhelming • Challenges in contacting relatives for residents lacking capacity within the study period • Hesitancy of healthcare professionals and CH staff to act as legal representatives	• Use short, illustrated information sheets with simple language and clear layout • Consent discussions without excess detail • Engage relatives regularly to build familiarity and trust • Senior CH staff or physicians to act as legal representatives • Present study risks and benefits in simple language to residents and CH staff to aid understanding and consent
	**Ethical and regulatory considerations**	
	• Placebo groups are not accepted when approved treatment exist • Delays in ethical approval for including adults lacking capacity in CTIMPs • Lack of clear research governance or regulatory processes for CHs as non-hospital trial sites • Separate approval needed if GPs are added as additional sites to CHs	• Provide scientific justification for placebo use and ensure standard of care for all participants • Develop a research governance toolkit with resources for conducting CH trials.
Trial cost	**Insurance and trial costs**	
	• Healthcare organisation indemnity does not cover CHs • Requirement for separate CH indemnity limit participation • Inadequate GPs reimbursement hinders involvement • High screen failure and dropouts in CH populations increase trial costs	• Offer small gifts to residents • Provide stipends to support CH staff • Use letters of agreements instead of formal contracts • Include indemnity costs in funding applications
Trial results	**Generalisability of trial results**	
	• Recruitment bias limits the generalisability and validity of trial findings	• Adjust sample size to account for reduced treatment effects in CH populations.

Abbreviations: CH: Care Home; GPs: General Practitioners/General Practices; CTIMPs: Clinical Trials of Investigational Medicinal Products.

One study reported that some relatives may make decisions based on their own preferences rather than residents’ wishes. Mistrust, anxiety about resident’s wellbeing, not wanting to be bothered, and geographical distance often limited relatives’ involvement as consultees or legal representatives. Both residents and relatives often struggled to understand study aims, and even residents with capacity had difficulty retaining information over time [31].

Despite these barriers, many residents were motivated to participate by altruism or a desire to help the wider community [31]. Two studies reported that using simple, non-technical language and summarising benefits and risks without quantifying individual risk can improve understanding and support informed decision-making [25, 26]. To reduce refusals, one trial employed informal capacity assessments [26], and another study recommended capacity assessment by senior CH staff [31].

#### CH facility and staff-related factors

CH professionals including owners, directors and managers have diverse clinical backgrounds and research experience, which can facilitate or hinder research engagement [27]. Recruiting CHs was highly challenging and time-consuming in two trials, taking 3 to 13 months [25, 27]. To recruit CHs, two trials adopted a multi-step approach: initial meetings with CH professionals to explain the study, followed by letters to affiliated physicians to confirm their participation, and obtaining institutional permission before approaching residents [25, 27]. A COVID-19 trial in the USA recruited CHs using a pre-existing list of contracted facilities [29].

CH staff often experience high workloads, and additional tasks of obtaining consent and completing study assessments can be burdensome, contributing to high staff turnover [25, 31]. Cultural factors, including ethnic diversity and language barriers may further complicate consent and understanding of research [26]. To reduce staff burden, two trials employed research teams to conduct study procedures which facilitated collaboration [25, 27]. One study found CH staff were motivated by training opportunities and professional benefits from participating in university-led research [31].

#### Healthcare professional involvement

Each participating CH requires a Principal Investigator to oversee trial conduct. In the UK, recruiting GPs as trial sites or research partners posed enormous practical challenges in one trial as CHs often receive care from multiple practices, complicating agreements, regulatory approval, and training [27]. Although some GPs were hesitant to include adults lacking capacity, most reported that they would support CH residents participating in research [31].

To overcome these challenges, one trial recruited general practices as research sites alongside CHs, assigning GPs responsibility for medical decisions and overall trial conduct. Furthermore, the trial implemented strategies (e.g. online training) to reduce GPs burden and encourage their involvement [27]. Additionally, studies reported that training physicians to raise awareness on CH trials, and training researchers in effective communication with older adults can improve recruitment and the presentation of complex trial information [31, 32]. One trial involved a multidisciplinary trial team of geriatricians, endocrinologists, nurses, research coordinators and long-term care specialists to support trial conduct in CH settings [25].

#### Logistical and operational factors

Transportation challenges and lack of essential research infrastructure such as laboratories, pharmacies, and dedicated assessment or treatment rooms often limit CH residents’ participation in clinical trials [25, 29]. To address this, two trials employed MRUs which were custom designed, equipped, and staffed to conduct study visits onsite at CH facilities. Functioning as extensions of investigator sites, MRUs brought clinical research directly to CH residents and staff and were designed to operate under extreme temperature conditions [25, 29].

#### Consent-related factors

Residents often found the detailed consent process overwhelming [25] and complex terminology in information sheets demotivated participation, though some found the information insufficient [31]. Many residents struggled to understand or retain study information, raising concerns about consent validity, particularly as some relatives may also experience cognitive decline [31]. Recruitment was limited when relatives of residents lacking capacity could not be contacted within the study timeframe [26, 31]. Other barriers included reluctance of healthcare professionals and staff to act as legal representatives and insufficient training among CH staff to understand trial risks and benefits [26, 31].

Recommendations to improve consent included using short, illustrated information sheets, with simple language, and clear layouts, and improving consent discussions without overwhelming residents with excessive detail [31]. One trial implemented a two-stage approach: initial consent for screening, followed by consent for randomization with eligible residents [25]. Relatives who regularly visited CHs and knew the residents were more likely to act as legal representatives [31]. When relatives were unavailable, senior CH staff familiar with residents’ preferences could act as nominated consultees [26, 31]. Agreement with CH physicians to provide power of attorney at residents’ request were also suggested to facilitate recruitment [25]. Presenting study risks and benefits in simple English could further help relatives and CH staff better understand the research when providing consent on residents’ behalf [26].

#### Ethical and regulatory considerations

Including a placebo group in a trial where approved treatments exist presented significant challenges for ethics approval. This trial scientifically justified its use and provided standard care to all participants [25]. In one probiotic trial, the regulatory authority classified the food supplement as a Clinical Trial of an Investigational Medicinal Product (CTIMP), causing a four-month delay due to repeated ethics requests to justify including adults lacking capacity. Further delays occurred when regulatory documents for the investigational product, usually unavailable for food supplements were required. Research governance in CHs is complex, involving commercial and non-commercial organisations. Adding general practices as sites in addition to CHs required separate approvals [27]. Developing a research governance toolkit for CH research would be beneficial [27].

#### Insurance and trial costs

Healthcare organisation indemnity does not extend to CHs, requiring separate arrangements that can delay trial setup and may limit CH from participation [27]. Inadequate GP reimbursement can further hinder their involvement [31]. Trials involving frail CH residents also incur hidden trial costs from high exclusion rates, screen failures, and consent difficulties [30]. To improve recruitment and retention, one trial provided small gift cards to residents and stipends to support CH staff salaries or purchases for CHs, as tokens of appreciation for their time and participation [25]. To minimise administrative burden, one trial used a letter of agreement with GPs in lieu of formal contracts [27]. They also recommended inclusion of indemnity costs in future funding applications [27].

#### Generalisability of trial results

Extremely low recruitment rates in dementia trials and differences between participants and non-participants can affect generalisability of results [30]. In one trial, difficulty contacting relatives for consent introduced recruitment bias, and overrepresented residents with capacity. This may undermine validity, as vaccine efficacy may differ in those with dementia [26]. Considering this, one trial adjusted sample size calculations by assuming a 25% reduction in treatment effect compared with published estimates [25].

## Discussion

Although previous reviews have reported barriers and facilitators to conducting research in CH settings, this is the first review to specifically identify common barriers and facilitators to recruiting CH residents into RCTs of pharmacological interventions. Six of the eight included studies reported the direct experiences of trial teams in conducting RCTs in CHs, providing valuable insights into practical recruitment challenges and innovative strategies implemented to address them.

We identified 13 key themes of barriers and facilitators for recruiting and retaining CH residents in RCTs of medicines and vaccines. The National Institute for Health and Care Research’s (NIHR) INCLUDE framework has provided recommendations for including older adults in health and social care research. These are not specific to RCTs but several of their recommendations align with our identified themes [33]. Two trials [25, 26] reported a screen failure rate of 70%, with one dementia trial [30] reporting an exceptionally high rate of 96%. Although the number of included studies was small, the main reasons for screen failure were refusal by residents or relatives, and the unavailability of relatives for residents lacking capacity to consent. Our review also highlights that exclusion of large proportions of residents with memory impairment introduces recruitment bias, limiting the generalisability of trial findings.

To improve the representation of CH residents in RCTs, strategies such as simplifying the consent process and obtaining consent through personal consultees or legal representatives may be effective [31, 34]. The NIHR INCLUDE Impaired Capacity to Consent Framework provides guidance for designing more inclusive trials involving adults with impaired capacity [35, 36]. Trials should also consider the high prevalence of sensory loss and physical dependency in CH populations [37]. It is also essential that trials measure outcomes that are clinically relevant and meaningful for this group [3, 38]. Use of multiple data sources and MRUs to conduct trials within CH settings has proven successful [25, 29]. The US Long-Term Care Data Cooperative that provides linked CH data could support trial planning and data collection [39]. CH professionals are generally supportive of research involving CH residents. However, research may not be a priority for overstretched CH staff [40]. Placing research teams within CHs, fostering collaboration with CHs and GPs, and offering incentives are crucial for the successful conduct of trials.

Although barriers identified are common to CH trials globally, some are specific to individual countries, states or institutions. Clinical trial regulations and legal and administrative requirements governing proxy consent, insurance, indemnity, data-sharing, and ethical and regulatory approvals vary across jurisdictions. These challenges require careful consideration and tailored guidance when planning international trials in CHs [7, 8, 11, 27, 41–44]. As non-hospital organisations, CHs in the UK currently lack the infrastructure, capacity, resources, and clear research governance, ethical approval, and regulatory processes, and contracting arrangements needed to conduct both non-CTIMPs and CTIMPs in CH settings [43–45] [42, 45].

CH research networks exist in the UK, USA, the Netherlands, and Canada., However, their scope and structure vary considerably, and CHs internationally were unprepared to conduct trials during the COVID-19 pandemic [7, 40, 43, 46, 47]. Post-pandemic, the USA has established new initiatives to develop CH research networks [7, 48] and the Netherlands ‘Living Lab’ model is being adopted in the UK, Germany and Austria [49]. Although CH research networks support research in CH settings, a collaborative, multi-stakeholder approach involving geriatricians, GPs, CH researchers, CH research networks, CH professionals, residents and relatives, regulatory bodies and the pharmaceutical industry is essential to build the infrastructure and capacity needed for CTIMPs in CHs and ensure preparedness for future pandemics [11, 40, 50].

### Strengths and limitations

The main strength of our review is that we focused exclusively on the barriers and facilitators to recruiting CH residents to RCTs of pharmacological interventions. We also followed the methodology recommended by the Joanna Briggs Institute and used the search strategy developed following an iterative process among the authors and input from the WATCH project Advisory Group, comprising experts in clinical trials and CH research, vaccine trials and geriatricians.

There are some limitations. In line with scoping review methodology, the quality of the included articles was not assessed. Although we used a comprehensive search strategy, some relevant articles may have been missed. To mitigate this, we performed reference checking and forward citation searching of the included articles to identify any additional relevant articles. Additionally, we excluded RCTs of pharmacological interventions that did not explicitly report barriers or facilitators to recruiting CH residents which may have influenced the screen failure rates reported in our review. Nevertheless, the low number of included articles is not surprising and highlights the paucity of RCTs of pharmacological interventions in CH populations.

## Conclusion

Our scoping review identified themes that could improve the recruitment and retention of CH residents into clinical trials of pharmacological interventions. Despite many existing recruitment challenges, conducting such trials in the CH population is feasible. Enhancing the inclusion of CH residents in trials may improve the representation and generalisability of trial findings, thereby supporting evidence-based care for this vulnerable population.

## Supplementary Material

Supplementary_Data_File_afag077
