# Supplementary material for: Barriers and facilitators to recruiting older adult care home residents into clinical trials of medicines and vaccines: a scoping review
Source: Age Ageing. 2026 Apr 9;55(4):afag077. doi: 10.1093/ageing/afag077 (PMC13070390; doi:10.1093/ageing/afag077)
Supplement: Supplementary_Data_File_afag077 [file supplementary_data_file_afag077.docx]

**Supplementary Data File**

**Title:** Barriers and facilitators to recruiting older adult care home residents into clinical trials of medicines and vaccines: a scoping review

**Contents**

[Appendix 1. Preferred Reporting Items for Systematic reviews and Meta-Analyses extension for Scoping Reviews (PRISMA-ScR) Checklist 2](#_Toc225448056)

[Appendix 2: Database search results 4](#_Toc225448057)

[Supplementary Table S1. Search results from EMBASE (Ovid) 4](#_Toc225448058)

[Supplementary Table S2. Search results from MEDLINE (Ovid) 6](#_Toc225448059)

[Supplementary Table S3. Search results from PsycINFO (Ovid) 8](#_Toc225448060)

[Supplementary Table S4. Search results from CINAHL (EBSCOhost) 11](#_Toc225448061)

[Supplementary Table S5. Search results from the Cochrane Library 14](#_Toc225448062)

[Appendix 3: Data Extraction Form 16](#_Toc225448063)

# Appendix 1. Preferred Reporting Items for Systematic reviews and Meta-Analyses extension for Scoping Reviews (PRISMA-ScR) Checklist

| **SECTION** | **ITEM** | **PRISMA-ScR CHECKLIST ITEM** | **REPORTED ON PAGE #** |
| --- | --- | --- | --- |
| **TITLE** | | | |
| Title | 1 | Identify the report as a scoping review. | 1 |
| **ABSTRACT** | | | |
| Structured summary | 2 | Provide a structured summary that includes (as applicable): background, objectives, eligibility criteria, sources of evidence, charting methods, results, and conclusions that relate to the review questions and objectives. | 1 |
| **INTRODUCTION** | | | |
| Rationale | 3 | Describe the rationale for the review in the context of what is already known. Explain why the review questions/objectives lend themselves to a scoping review approach. | 2 |
| Objectives | 4 | Provide an explicit statement of the questions and objectives being addressed with reference to their key elements (e.g., population or participants, concepts, and context) or other relevant key elements used to conceptualize the review questions and/or objectives. | 2 |
| **METHODS** | | | |
| Protocol and registration | 5 | Indicate whether a review protocol exists; state if and where it can be accessed (e.g., a Web address); and if available, provide registration information, including the registration number. | 2 |
| Eligibility criteria | 6 | Specify characteristics of the sources of evidence used as eligibility criteria (e.g., years considered, language, and publication status), and provide a rationale. | 2 |
| Information sources | 7 | Describe all information sources in the search (e.g., databases with dates of coverage and contact with authors to identify additional sources), as well as the date the most recent search was executed. | 2 |
| Search | 8 | Present the full electronic search strategy for at least 1 database, including any limits used, such that it could be repeated. | 2 & Appendix 2 |
| Selection of sources of evidence | 9 | State the process for selecting sources of evidence (i.e., screening and eligibility) included in the scoping review. | 2-3 |
| Data charting process | 10 | Describe the methods of charting data from the included sources of evidence (e.g., calibrated forms or forms that have been tested by the team before their use, and whether data charting was done independently or in duplicate) and any processes for obtaining and confirming data from investigators. | 3 |
| Data items | 11 | List and define all variables for which data were sought and any assumptions and simplifications made. | 3 & Appendix 3 |
| Critical appraisal of individual sources of evidence | 12 | If done, provide a rationale for conducting a critical appraisal of included sources of evidence; describe the methods used and how this information was used in any data synthesis (if appropriate). | Not done |
| Synthesis of results | 13 | Describe the methods of handling and summarizing the data that were charted. | 3 |
| **RESULTS** | | | |
| Selection of sources of evidence | 14 | Give numbers of sources of evidence screened, assessed for eligibility, and included in the review, with reasons for exclusions at each stage, ideally using a flow diagram. | 3 & Figure 1 |
| Characteristics of sources of evidence | 15 | For each source of evidence, present characteristics for which data were charted and provide the citations. | 3-4 &  Table 1 |
| Critical appraisal within sources of evidence | 16 | If done, present data on critical appraisal of included sources of evidence (see item 12). | Not done |
| Results of individual sources of evidence | 17 | For each included source of evidence, present the relevant data that were charted that relate to the review questions and objectives. | 4-9 |
| Synthesis of results | 18 | Summarize and/or present the charting results as they relate to the review questions and objectives. | 4-9,  Figure 2 &  Table 2 & 3 |
| **DISCUSSION** | | | |
| Summary of evidence | 19 | Summarize the main results (including an overview of concepts, themes, and types of evidence available), link to the review questions and objectives, and consider the relevance to key groups. | 9 to 10 |
| Limitations | 20 | Discuss the limitations of the scoping review process. | 10 |
| Conclusions | 21 | Provide a general interpretation of the results with respect to the review questions and objectives, as well as potential implications and/or next steps. | 10 |
| **FUNDING** | | | |
| Funding | 22 | Describe sources of funding for the included sources of evidence, as well as sources of funding for the scoping review. Describe the role of the funders of the scoping review. | 11 |

# Appendix 2: Database search results

## Supplementary Table S1. Search results from EMBASE (Ovid)

| [#](https://ovidsp.dc1.ovid.com/ovid-new-b/ovidweb.cgi?&S=GEMPFPDDMBACDHKJKPIJLENMPELGAA00&Sort+Sets=descending) | Searches | Results |
| --- | --- | --- |
| 1 | exp aged/ | 4189068 |
| 2 | exp aging/ | 367466 |
| 3 | ageing.tw. | 77320 |
| 4 | elder*.tw. | 467312 |
| 5 | exp frail elderly/ | 12945 |
| 6 | older people.tw. | 51287 |
| 7 | older adult*.tw. | 172691 |
| 8 | older population.tw. | 10631 |
| 9 | older individual*.tw. | 18319 |
| 10 | oldest old.tw. | 4169 |
| 11 | exp geriatrics/ | 61926 |
| 12 | geriatric*.tw. | 107176 |
| 13 | very elderly/ | 334206 |
| 14 | barriers.tw. | 283833 |
| 15 | facilitators.tw. | 40044 |
| 16 | enablers.tw. | 7319 |
| 17 | advantages.tw. | 403586 |
| 18 | disadvantages.tw. | 78385 |
| 19 | challenges.tw. | 638925 |
| 20 | solutions.tw. | 374565 |
| 21 | burden.tw. | 524854 |
| 22 | recruit*.tw. | 796092 |
| 23 | retention.tw. | 309192 |
| 24 | enrol*.tw. | 948572 |
| 25 | participat*.tw. | 996069 |
| 26 | research participation.tw. | 2846 |
| 27 | inclusion.tw. | 567492 |
| 28 | perception*.tw. | 444211 |
| 29 | perspective*.tw. | 608802 |
| 30 | views.tw. | 137307 |
| 31 | opinion.tw. | 144424 |
| 32 | preference*.tw. | 263668 |
| 33 | lessons.tw. | 103995 |
| 34 | beliefs.tw. | 95687 |
| 35 | attitudes.tw. | 196968 |
| 36 | experience*.tw. | 2183296 |
| 37 | factors.tw. | 3800439 |
| 38 | issues.tw. | 571758 |
| 39 | difficult*.tw. | 1201481 |
| 40 | recommendations.tw. | 459566 |
| 41 | strateg*.tw. | 2172621 |
| 42 | care home*.tw. | 8089 |
| 43 | nursing home/ | 68598 |
| 44 | old age home*.tw. | 563 |
| 45 | aged care home*.tw. | 225 |
| 46 | residential home/ | 8643 |
| 47 | residential care.tw. | 5670 |
| 48 | residential facilit*.tw. | 1555 |
| 49 | long term care.tw. | 33840 |
| 50 | long-term care setting*.tw. | 2087 |
| 51 | long-term care facilit*.tw. | 9709 |
| 52 | care home setting*.tw. | 317 |
| 53 | home for the aged/ | 13919 |
| 54 | aged care facilit*.tw. | 1877 |
| 55 | institutional care/ | 6912 |
| 56 | institutionalization/ | 9999 |
| 57 | institutionali?ation.tw. | 8208 |
| 58 | institutionalized adult/ or institutionalized elderly/ | 470 |
| 59 | institutionali?ed.tw. | 16966 |
| 60 | randomized controlled trial/ | 862368 |
| 61 | randomi?ed controlled trial.tw. | 199386 |
| 62 | RCT.tw. | 60921 |
| 63 | controlled clinical trial/ | 442539 |
| 64 | controlled trial.tw. | 254468 |
| 65 | clinical trial/ | 1106455 |
| 66 | trial*.tw. | 2091656 |
| 67 | clinical study/ | 181278 |
| 68 | randomi?ed.tw. | 1245802 |
| 69 | randomization/ | 99780 |
| 70 | random allocation.tw. | 2834 |
| 71 | randomly allocated.tw. | 49545 |
| 72 | random assignment.tw. | 3553 |
| 73 | 1 or 2 or 3 or 4 or 5 or 6 or 7 or 8 or 9 or 10 or 11 or 12 or 13 | 4629548 |
| 74 | 14 or 15 or 16 or 17 or 18 or 19 or 20 or 21 or 22 or 23 or 24 or 25 or 26 or 27 or 28 or 29 or 30 or 31 or 32 or 33 or 34 or 35 or 36 or 37 or 38 or 39 or 40 or 41 | 12983781 |
| 75 | 60 or 61 or 62 or 63 or 64 or 65 or 66 or 67 or 68 or 69 or 70 or 71 or 72 | 3413669 |
| 76 | 42 or 43 or 44 or 45 or 46 or 47 or 48 or 49 or 50 or 51 or 52 or 53 or 54 or 55 or 56 or 57 or 58 or 59 | 146251 |
| 77 | 73 and 74 and 75 and 76 | 4807 |
| 78 | limit 77 to yr="1990 -Current" | 4763 |

## Supplementary Table S2. Search results from MEDLINE (Ovid)

| \| # \| Searches \| Results \| \| --- \| --- \| --- \| \| 1 \| exp Aged/ \| 3629201 \| \| 2 \| exp Aging/ \| 305511 \| \| 3 \| ageing.tw. \| 57061 \| \| 4 \| elder*.tw. \| 323818 \| \| 5 \| exp Frail Elderly/ \| 16840 \| \| 6 \| older people.tw. \| 41609 \| \| 7 \| older adult*.tw. \| 141319 \| \| 8 \| older population.tw. \| 7675 \| \| 9 \| older individual*.tw. \| 14493 \| \| 10 \| oldest old.tw. \| 3291 \| \| 11 \| exp Geriatrics/ \| 32069 \| \| 12 \| geriatric*.tw. \| 64227 \| \| 13 \| "Aged, 80 and over"/ \| 1059115 \| \| 14 \| barriers.tw. \| 227737 \| \| 15 \| facilitators.tw. \| 32718 \| \| 16 \| enablers.tw. \| 6233 \| \| 17 \| advantages.tw. \| 319421 \| \| 18 \| disadvantages.tw. \| 59844 \| \| 19 \| challenges.tw. \| 551135 \| \| 20 \| solutions.tw. \| 322178 \| \| 21 \| burden.tw. \| 342912 \| \| 22 \| recruit*.tw. \| 546753 \| \| 23 \| retention.tw. \| 235035 \| \| 24 \| enrol*.tw. \| 538236 \| \| 25 \| participat*.tw. \| 746995 \| \| 26 \| research participation.tw. \| 2129 \| \| 27 \| inclusion.tw. \| 378717 \| \| 28 \| perception*.tw. \| 364409 \| \| 29 \| perspective*.tw. \| 522049 \| \| 30 \| views.tw. \| 101090 \| \| 31 \| opinion.tw. \| 100127 \| \| 32 \| preference*.tw. \| 212144 \| \| 33 \| lessons.tw. \| 87174 \| \| 34 \| beliefs.tw. \| 79643 \| \| 35 \| attitudes.tw. \| 160788 \| \| 36 \| experience*.tw. \| 1510353 \| \| 37 \| factors.tw. \| 2825772 \| \| 38 \| issues.tw. \| 445793 \| \| 39 \| difficult*.tw. \| 847393 \| \| 40 \| recommendations.tw. \| 339535 \| \| 41 \| strateg*.tw. \| 1771153 \| \| 42 \| Randomized Controlled Trial/ \| 630208 \| \| 43 \| randomi?ed controlled trial.tw. \| 156081 \| \| 44 \| RCT.tw. \| 36753 \| \| 45 \| Controlled Clinical Trial/ \| 95676 \| \| 46 \| controlled trial.tw. \| 195456 \| \| 47 \| Clinical Trial/ \| 540985 \| \| 48 \| trial*.tw. \| 1462185 \| \| 49 \| Clinical Study/ \| 6504 \| \| 50 \| randomi?ed.tw. \| 876249 \| \| 51 \| randomi?ation.tw. \| 63661 \| \| 52 \| Random Allocation/ \| 107993 \| \| 53 \| randomly allocated.tw. \| 40403 \| \| 54 \| random assignment.tw. \| 2973 \| \| 55 \| care home*.tw. \| 6072 \| \| 56 \| Nursing Homes/ \| 40865 \| \| 57 \| old age home*.tw. \| 367 \| \| 58 \| aged care home*.tw. \| 204 \| \| 59 \| residential home*.tw. \| 1102 \| \| 60 \| residential care.tw. \| 4573 \| \| 61 \| Residential Facilities/ \| 5810 \| \| 62 \| residential facilit*.tw. \| 1214 \| \| 63 \| Long-Term Care/ \| 29392 \| \| 64 \| long-term care setting*.tw. \| 1697 \| \| 65 \| long-term care facilit*.tw. \| 7551 \| \| 66 \| care home setting*.tw. \| 202 \| \| 67 \| Homes for the Aged/ \| 15245 \| \| 68 \| aged care facilit*.tw. \| 1661 \| \| 69 \| institutional care.tw. \| 2348 \| \| 70 \| Institutionalization/ \| 5577 \| \| 71 \| institutionali?ation.tw. \| 6005 \| \| 72 \| institutionali?ed.tw. \| 12573 \| \| 73 \| 1 or 2 or 3 or 4 or 5 or 6 or 7 or 8 or 9 or 10 or 11 or 12 or 13 \| 4003360 \| \| 74 \| 14 or 15 or 16 or 17 or 18 or 19 or 20 or 21 or 22 or 23 or 24 or 25 or 26 or 27 or 28 or 29 or 30 or 31 or 32 or 33 or 34 or 35 or 36 or 37 or 38 or 39 or 40 or 41 \| 9730157 \| \| 75 \| 42 or 43 or 44 or 45 or 46 or 47 or 48 or 49 or 50 or 51 or 52 or 53 or 54 \| 2268960 \| \| 76 \| 55 or 56 or 57 or 58 or 59 or 60 or 61 or 62 or 63 or 64 or 65 or 66 or 67 or 68 or 69 or 70 or 71 or 72 \| 104790 \| \| 77 \| 73 and 74 and 75 and 76 \| 3377 \| \| 78 \| limit 77 to yr="1990 -Current" \| 3310 \| |
| --- | --- | --- | --- | --- | --- | --- | --- | --- | --- | --- | --- | --- | --- | --- | --- | --- | --- | --- | --- | --- | --- | --- | --- | --- | --- | --- | --- | --- | --- | --- | --- | --- | --- | --- | --- | --- | --- | --- | --- | --- | --- | --- | --- | --- | --- | --- | --- | --- | --- | --- | --- | --- | --- | --- | --- | --- | --- | --- | --- | --- | --- | --- | --- | --- | --- | --- | --- | --- | --- | --- | --- | --- | --- | --- | --- | --- | --- | --- | --- | --- | --- | --- | --- | --- | --- | --- | --- | --- | --- | --- | --- | --- | --- | --- | --- | --- | --- | --- | --- | --- | --- | --- | --- | --- | --- | --- | --- | --- | --- | --- | --- | --- | --- | --- | --- | --- | --- | --- | --- | --- | --- | --- | --- | --- | --- | --- | --- | --- | --- | --- | --- | --- | --- | --- | --- | --- | --- | --- | --- | --- | --- | --- | --- | --- | --- | --- | --- | --- | --- | --- | --- | --- | --- | --- | --- | --- | --- | --- | --- | --- | --- | --- | --- | --- | --- | --- | --- | --- | --- | --- | --- | --- | --- | --- | --- | --- | --- | --- | --- | --- | --- | --- | --- | --- | --- | --- | --- | --- | --- | --- | --- | --- | --- | --- | --- | --- | --- | --- | --- | --- | --- | --- | --- | --- | --- | --- | --- | --- | --- | --- | --- | --- | --- | --- | --- | --- | --- | --- | --- | --- | --- | --- | --- | --- | --- | --- | --- | --- | --- | --- | --- | --- | --- | --- | --- | --- | --- |

## Supplementary Table S3. Search results from PsycINFO (Ovid)

| # | Searches | Results |
| --- | --- | --- |
| 1 | aged.tw. | 286787 |
| 2 | exp Aging/ | 94505 |
| 3 | ageing.tw. | 13925 |
| 4 | elder*.tw. | 74892 |
| 5 | elderly.tw. | 64822 |
| 6 | frail elderly.tw. | 1041 |
| 7 | older people.tw. | 18007 |
| 8 | older adult*.tw. | 72521 |
| 9 | older population.tw. | 2207 |
| 10 | older individual*.tw. | 4445 |
| 11 | oldest old.tw. | 1506 |
| 12 | exp Geriatrics/ | 15205 |
| 13 | geriatric*.tw. | 19635 |
| 14 | very elderly.tw. | 211 |
| 15 | exp Randomized Controlled Trials/ | 1712 |
| 16 | randomi?ed controlled trial.tw. | 33193 |
| 17 | RCT.tw. | 7793 |
| 18 | controlled clinical trial.tw. | 1955 |
| 19 | controlled trial.tw. | 38834 |
| 20 | exp Clinical Trials/ | 14079 |
| 21 | trial*.tw. | 210297 |
| 22 | clinical study.tw. | 2425 |
| 23 | exp Randomized Clinical Trials/ | 591 |
| 24 | randomi?ed.tw. | 116386 |
| 25 | randomi?ation.tw. | 7619 |
| 26 | random allocation.tw. | 320 |
| 27 | randomly allocated.tw. | 4679 |
| 28 | random assignment.tw. | 2102 |
| 29 | care home*.tw. | 2532 |
| 30 | exp Nursing Homes/ | 10048 |
| 31 | old age home*.tw. | 149 |
| 32 | aged care home*.tw. | 89 |
| 33 | residential home*.tw. | 600 |
| 34 | residential care.tw. | 4864 |
| 35 | exp Residential Care Institutions/ | 45671 |
| 36 | residential facilit*.tw. | 1480 |
| 37 | exp Long Term Care/ | 7086 |
| 38 | long-term care setting*.tw. | 829 |
| 39 | long-term care facilit*.tw. | 2060 |
| 40 | care home setting*.tw. | 79 |
| 41 | homes for the aged.tw. | 157 |
| 42 | aged care facilit*.tw. | 621 |
| 43 | institutional care.tw. | 1667 |
| 44 | exp Institutionalization/ | 44619 |
| 45 | institutionali?ation.tw. | 5171 |
| 46 | institutionali?ed.tw. | 8505 |
| 47 | barriers.tw. | 87083 |
| 48 | facilitators.tw. | 15628 |
| 49 | enablers.tw. | 2455 |
| 50 | advantages.tw. | 35378 |
| 51 | disadvantages.tw. | 10246 |
| 52 | challenges.tw. | 185382 |
| 53 | solutions.tw. | 42928 |
| 54 | burden.tw. | 52796 |
| 55 | recruit*.tw. | 134656 |
| 56 | retention.tw. | 45880 |
| 57 | enrol*.tw. | 75234 |
| 58 | participat*.tw. | 325382 |
| 59 | research participation.tw. | 1422 |
| 60 | inclusion.tw. | 78517 |
| 61 | perception*.tw. | 353062 |
| 62 | perspective*.tw. | 347850 |
| 63 | views.tw. | 73673 |
| 64 | opinion.tw. | 26855 |
| 65 | preference*.tw. | 107174 |
| 66 | lessons.tw. | 36083 |
| 67 | beliefs.tw. | 115436 |
| 68 | attitudes.tw. | 189939 |
| 69 | experience*.tw. | 772213 |
| 70 | factors.tw. | 631207 |
| 71 | issues.tw. | 315397 |
| 72 | difficult*.tw. | 261833 |
| 73 | recommendations.tw. | 120720 |
| 74 | strateg*.tw. | 422676 |
| 75 | 1 or 2 or 3 or 4 or 5 or 6 or 7 or 8 or 9 or 10 or 11 or 12 or 13 or 14 | 446653 |
| 76 | 15 or 16 or 17 or 18 or 19 or 20 or 21 or 22 or 23 or 24 or 25 or 26 or 27 or 28 | 250090 |
| 77 | 29 or 30 or 31 or 32 or 33 or 34 or 35 or 36 or 37 or 38 or 39 or 40 or 41 or 42 or 43 or 44 or 45 or 46 | 106335 |
| 78 | 47 or 48 or 49 or 50 or 51 or 52 or 53 or 54 or 55 or 56 or 57 or 58 or 59 or 60 or 61 or 62 or 63 or 64 or 65 or 66 or 67 or 68 or 69 or 70 or 71 or 72 or 73 or 74 | 2797771 |
| 79 | 75 and 76 and 77 and 78 | 840 |
| 80 | limit 79 to yr="1990 -Current" | 837 |
|  |  |  |

## Supplementary Table S4. Search results from CINAHL (EBSCOhost)

| [**Search ID#**](javascript:__doPostBack('ctl00$ctl00$FindField$FindField$historyControl$ReorderHistoryLink','')) | **Search Terms** | **Actions** |
| --- | --- | --- |
| S1 | (MH "Aged") | 945,456 |
| S2 | aged | 1,135,614 |
| S3 | (MH "Aging") | 56,967 |
| S4 | aging | 114,016 |
| S5 | ageing | 114,016 |
| S6 | elder | 16,037 |
| S7 | elders | 17,469 |
| S8 | elderly | 113,149 |
| S9 | (MH "Frail Elderly") | 9,079 |
| S10 | frail elderly | 10,304 |
| S11 | older people | 35,810 |
| S12 | older adults | 97,707 |
| S13 | older population | 15,777 |
| S14 | older individuals | 10,969 |
| S15 | oldest old | 2,168 |
| S16 | (MH "Geriatrics") | 6,221 |
| S17 | geriatric | 56,254 |
| S18 | (MH "Aged, 80 and Over") | 345,796 |
| S19 | aged 80 and over | 345,923 |
| S20 | barriers | 132,055 |
| S21 | facilitators | 23,085 |
| S22 | advantages | 63,996 |
| S23 | disadvantages | 17,623 |
| S24 | solutions | 89,295 |
| S25 | recruitment | 58,302 |
| S26 | recruiting | 12,824 |
| S27 | recruitment strategies | 2,848 |
| S28 | enrollment | 21,640 |
| S29 | enrolled | 135,276 |
| S30 | participation | 129,811 |
| S31 | participating | 46,417 |
| S32 | participate | 52,792 |
| S33 | research participation | 4,721 |
| S34 | inclusion | 120,785 |
| S35 | perception | 203,435 |
| S36 | perspective | 182,802 |
| S37 | views | 108,476 |
| S38 | opinion | 51,809 |
| S39 | preference | 61,776 |
| S40 | lessons | 40,640 |
| S41 | beliefs | 68,038 |
| S42 | TI attitudes | 38,208 |
| S43 | TI experiences | 133,018 |
| S44 | TI factors | 193,379 |
| S45 | TI issues | 79,911 |
| S46 | difficulties | 101,687 |
| S47 | recommendations | 146,560 |
| S48 | TI (strategy or strategies) | 67,286 |
| S49 | enablers | 4,044 |
| S50 | retention | 46,373 |
| S51 | (MH "Randomized Controlled Trials") | 147,767 |
| S52 | randomised controlled trial | 36,640 |
| S53 | rct | 32,519 |
| S54 | controlled clinical trial | 39,511 |
| S55 | controlled trial | 266,351 |
| S56 | (MH "Clinical Trials") | 187,148 |
| S57 | clinical trial | 306,273 |
| S58 | trial | 606,194 |
| S59 | clinical study | 148,351 |
| S60 | randomized | 328,780 |
| S61 | randomised | 59,154 |
| S62 | randomization | 23,262 |
| S63 | randomisation | 21,333 |
| S64 | random allocation | 1,338 |
| S65 | randomly allocated | 12,801 |
| S66 | (MH "Random Assignment") | 89,379 |
| S67 | random assignment | 90,426 |
| S68 | care home | 67,575 |
| S69 | (MH "Nursing Homes") | 26,824 |
| S70 | nursing home | 60,752 |
| S71 | old age home | 3,925 |
| S72 | aged care home | 651 |
| S73 | residential home | 2,360 |
| S74 | residential care | 14,068 |
| S75 | (MH "Residential Facilities") | 5,666 |
| S76 | residential facility | 7,571 |
| S77 | long term care | 43,359 |
| S78 | long term care setting | 2,448 |
| S79 | longterm care facility | 35 |
| S80 | care home setting | 2,941 |
| S81 | homes for the aged | 4,926 |
| S82 | aged care facility | 1,626 |
| S83 | institutional care | 5,014 |
| S84 | (MH "Institutionalization") | 2,848 |
| S85 | institutionalisation | 5,120 |
| S86 | institutionalized | 4,904 |
| S87 | TI challenges | 61,503 |
| S88 | TI burden | 23,437 |
| S89 | S1 OR S2 OR S3 OR S4 OR S5 OR S6 OR S7 OR S8 OR S9 OR S10 OR S11 OR S12 OR S13 OR S14 OR S15 OR S16 OR S17 OR S18 OR S19 | 1,250,248 |
| S90 | S20 OR S21 OR S22 OR S23 OR S24 OR S25 OR S26 OR S27 OR S28 OR S29 OR S30 OR S31 OR S32 OR S33 OR S34 OR S35 OR S36 OR S37 OR S38 OR S39 OR S40 OR S41 OR S42 OR S43 OR S44 OR S45 OR S46 OR S47 OR S48 OR S49 OR S50 OR S87 OR S88 | 1,883,213 |
| S91 | S51 OR S52 OR S53 OR S54 OR S55 OR S56 OR S57 OR S58 OR S59 OR S60 OR S61 OR S62 OR S63 OR S64 OR S65 OR S66 OR S67 | 764,604 |
| S92 | S68 OR S69 OR S70 OR S71 OR S72 OR S73 OR S74 OR S75 OR S76 OR S77 OR S78 OR S79 OR S80 OR S81 OR S82 OR S83 OR S84 OR S85 OR S86 | 158,620 |
| S93 | S89 AND S90 AND S91 AND S92 | 3,117 |
| S94 | S89 AND S90 AND S91 AND S92  Limiters- Publication Date: 19900101-20251231 | 3,110 |

## Supplementary Table S5. Search results from the Cochrane Library

| #1 | MeSH descriptor: [Aged] in all MeSH products | 283161 |
| --- | --- | --- |
| #2 | MeSH descriptor: [Frail Elderly] explode all trees | 1191 |
| #3 | MeSH descriptor: [Aged, 80 and over] explode all trees | 72289 |
| #4 | (aging):ti,ab,kw | 19908 |
| #5 | (ageing):ti,ab,kw | 19908 |
| #6 | (elderly):ti,ab,kw | 63404 |
| #7 | (older people):ti,ab,kw | 14371 |
| #8 | (older adults):ti,ab,kw | 28020 |
| #9 | (older population):ti,ab,kw | 13573 |
| #10 | (older individuals):ti,ab,kw | 8093 |
| #11 | (oldest old):ti,ab,kw | 186 |
| #12 | (geriatrics):ti,ab,kw | 1522 |
| #13 | #1 OR #2 OR #3 OR #4 OR #5 OR #6 OR #7 OR #8 OR #9 OR #10 OR #11 OR #12 | 358800 |
| #14 | MeSH descriptor: [Randomized Controlled Trial] explode all trees | 37 |
| #15 | ("randomised controlled trials"):ti | 6068 |
| #16 | ("randomised-controlled trial"):ti | 153340 |
| #17 | (RCT):ti,ab,kw | 43747 |
| #18 | MeSH descriptor: [Controlled Clinical Trial] explode all trees | 40 |
| #19 | (controlled trial):ti | 225976 |
| #20 | MeSH descriptor: [Clinical Trial] explode all trees | 45 |
| #21 | MeSH descriptor: [Clinical Study] explode all trees | 45 |
| #22 | (randomization OR randomisation):ti | 2254 |
| #23 | MeSH descriptor: [Random Allocation] explode all trees | 26097 |
| #24 | (randomly allocated):ti,ab,kw | 58048 |
| #25 | (random assignment):ti,ab,kw | 17394 |
| #26 | #14 OR #15 OR #16 OR #17 OR #18 OR #19 OR #20 OR #21 OR #22 OR #23 OR #24 OR #25 | 345459 |
| #27 | (care home*):ti,ab,kw | 34219 |
| #28 | MeSH descriptor: [Nursing Homes] explode all trees | 2140 |
| #29 | (old age home*):ti,ab,kw | 2407 |
| #30 | (aged care home):ti,ab,kw | 12626 |
| #31 | (residential home*):ti,ab,kw | 1245 |
| #32 | (residential care):ti,ab,kw | 2028 |
| #33 | MeSH descriptor: [Residential Facilities] explode all trees | 2676 |
| #34 | (residential facility):ti,ab,kw | 422 |
| #35 | MeSH descriptor: [Long-Term Care] explode all trees | 1553 |
| #36 | (long-term care setting*):ti,ab,kw | 5094 |
| #37 | (long-term care facilit*):ti,ab,kw | 3192 |
| #38 | (care home setting*):ti,ab,kw | 6672 |
| #39 | MeSH descriptor: [Homes for the Aged] explode all trees | 841 |
| #40 | (aged care facilit*):ti,ab,kw | 9055 |
| #41 | (institutional care):ti,ab,kw | 5305 |
| #42 | MeSH descriptor: [Institutionalization] explode all trees | 254 |
| #43 | (institutionalizations):ti,ab,kw | 21 |
| #44 | (institutionalisation*):ti,ab,kw | 159 |
| #45 | (institutionalized):ti,ab,kw | 1388 |
| #46 | (institutionalised):ti,ab,kw | 1388 |
| #47 | #27 OR #28 OR #29 OR #30 OR #31 OR #32 OR #33 OR #34 OR #35 OR #36 OR #37 OR #38 OR #39 OR #40 OR #41 OR #42 OR #43 OR #44 OR #45 OR #46 | 55240 |
| #48 | (barriers):ti,ab,kw | 18255 |
| #49 | (facilitators):ti,ab,kw | 4578 |
| #50 | (enablers):ti,ab,kw | 527 |
| #51 | (advantages):ti,ab,kw | 18620 |
| #52 | (disadvantages):ti,ab,kw | 3271 |
| #53 | (challenges):ti,ab,kw | 17524 |
| #54 | (solutions):ti,ab,kw | 20403 |
| #55 | (burden):ti,ab,kw | 34823 |
| #56 | ("recruitment"):ti,ab,kw | 37362 |
| #57 | (recruiting):ti,ab,kw | 6332 |
| #58 | (retention):ti,ab,kw | 28889 |
| #59 | (enrolment):ti,ab,kw | 45204 |
| #60 | (enrollment):ti,ab,kw | 45204 |
| #61 | (enroll*):ti | 2122 |
| #62 | (participation):ti,ab,kw | 46425 |
| #63 | (participate):ti,ab,kw | 70785 |
| #64 | (participat*):ti | 4594 |
| #65 | (research participation):ti,ab,kw | 13961 |
| #66 | (inclusion):ti | 572 |
| #67 | (perception*):ti,ab,kw | 42627 |
| #68 | (perspective*):ti | 3053 |
| #69 | (views):ti,ab,kw | 4847 |
| #70 | (opinion):ti,ab,kw | 7672 |
| #71 | (preference*):ti,ab,kw | 22491 |
| #72 | (lessons):ti,ab,kw | 5555 |
| #73 | (beliefs):ti,ab,kw | 8481 |
| #74 | (attitudes):ti | 2446 |
| #75 | (experience*):ti | 14668 |
| #76 | (factors):ti | 17458 |
| #77 | (issues):ti | 1006 |
| #78 | (difficult*):ti | 2523 |
| #79 | (recommendations):ti,ab,kw | 24868 |
| #80 | (strategy):ti | 6709 |
| #81 | (strategies):ti | 7522 |
| #82 | #48 OR #49 R #50 OR #51 OR #52 OR #53 OR #54 OR #55 OR #56 OR #57 OR #58 OR #59 OR #60 OR #61 OR #62 OR #63 OR #64 OR #65 OR #66 OR #67 OR #68 OR #69 OR #70 OR #71 OR #72 OR #73 OR #74 OR #75 OR #76 OR #77 OR #78 OR #79 OR #80 OR #81 | 405739 |
| #83 | #13 AND #26 AND #47 AND #82  with Cochrane Library publication date from Jan 1990 to Jan 2025 | 2281 |

# Appendix 3: Data Extraction Form

| **ADMINISTRATION DETAILS** | |
| --- | --- |
| Study ID | Last name of the first author and publication year |
| Publication status | e.g. full-text paper, conference abstract, trial registration |
| Language | Specify if its non-English language |
| Funding | Public or private funding |
| **STUDY CHARACTERISTICS** | |
| Study title and aim |  |
| Study design | e.g. Randomised Controlled Trial |
| Country | Country/countries where the study was conducted |
| Study population/Study setting | e.g. nursing home residents, long-term care facilities |
| Disease condition |  |
| Study period | Specify start and end date/year |
| Follow up period | Specify length of the follow up period |
| Eligibility Criteria | List of inclusion and exclusion criteria |
| **PARTICIPANT CHARACTERISTICS** | |
| Age (years) | Specify mean/median/range |
| Gender | n (%) for female |
| Race/Ethnicity | n (%) |
| **INTERVENTION AND COMPARATOR** | Details of intervention and comparator/placebo,  route of administration, storage/logistics,  licensed or Investigational Medicinal Product |
| **OUTCOMES REPORTED** | e.g. clinical outcomes, laboratory, adverse events if required and relevant |
| **CARE HOME CHARACTERISTICS** | |
| Number of Care homes | Number of care homes recruited participants |
| Type of care home | e.g. nursing home, residential home |
| Services provided | e.g. personal care, nursing care |
| Size of care home | Number of beds or small/medium/large |
| Ownership | e.g. public or private |
| **QUANTITATIVE DATA: SCREENING AND DROPOUT** | |
| Number screened | n (%) |
| Number recruited | n (%) |
| Number dropped out | n (%) |
| Number of screen failures | n (%) |
| Reasons for screen failures | List of reasons, specify if n (%) reported |
| Reasons for dropouts | List of reasons, specify if n (%) reported |
| **QUALITATIVE EVIDENCE OF BARRIERS AND FACILITATORS** | |
| Barriers reported | Barriers/challenges experienced by investigators in conducing clinical trials in care homes (e.g. recruitment, consent, regulatory issues, care home related factors etc) and views of different stakeholders |
| Facilitators reported | Strategies implemented by investigators to overcome the barriers (e.g. recruiting from multiple sites, change of study procedures, collaboration with care homes) and views of different stakeholders to facilitate clinical trials in care homes |
| **ETHICAL AND REGULATORY ASPECTS** | Ethical approval, consent, proxy consent, and gifts/incentives provided. Clinical trial registration |
